# Integration of Computational Docking into Anti-Cancer Drug Response Prediction Models

**DOI:** 10.3390/cancers16010050

**Published:** 2023-12-21

**Authors:** Oleksandr Narykov, Yitan Zhu, Thomas Brettin, Yvonne A. Evrard, Alexander Partin, Maulik Shukla, Fangfang Xia, Austin Clyde, Priyanka Vasanthakumari, James H. Doroshow, Rick L. Stevens

**Affiliations:** 1 Computing, Environment and Life Sciences, Argonne National Laboratory, Lemont, IL 60439, USA; yitan.zhu@anl.gov (Y.Z.); brettin@anl.gov (T.B.); apartin@anl.gov (A.P.); mshukla@anl.gov (M.S.); fangfang@anl.gov (F.X.); pvasanthakumari@anl.gov (P.V.); stevens@anl.gov (R.L.S.); 2 Leidos Biomedical Research, Frederick National Laboratory for Cancer Research, Frederick, MD 21702, USA; evrardy@mail.nih.gov; 3 Department of Computer Science, The University of Chicago, Chicago, IL 60637, USA; 4 Developmental Therapeutics Branch, National Cancer Institute, Bethesda, MD 20892, USA; doroshoj@mail.nih.gov

**Keywords:** anti-cancer drug response prediction, machine learning, deep learning, binding affinity, computational docking, molecular mechanisms of action

## Abstract

**Simple Summary:**

Anti-cancer drug response prediction models aim to reduce the time necessary for developing a treatment for patients affected by this complex disease. Their goal is to decrease the number of required biological experiments by computationally weeding out unpromising compounds. In this work, we explore the potential gains of incorporating large-scale applications of classical virtual screening techniques like molecular docking into cutting-edge deep learning models. We demonstrate improvement in performance as well as limitations of our approach.

**Abstract:**

Cancer is a heterogeneous disease in that tumors of the same histology type can respond differently to a treatment. Anti-cancer drug response prediction is of paramount importance for both drug development and patient treatment design. Although various computational methods and data have been used to develop drug response prediction models, it remains a challenging problem due to the complexities of cancer mechanisms and cancer-drug interactions. To better characterize the interaction between cancer and drugs, we investigate the feasibility of integrating computationally derived features of molecular mechanisms of action into prediction models. Specifically, we add docking scores of drug molecules and target proteins in combination with cancer gene expressions and molecular drug descriptors for building response models. The results demonstrate a marginal improvement in drug response prediction performance when adding docking scores as additional features, through tests on large drug screening data. We discuss the limitations of the current approach and provide the research community with a baseline dataset of the large-scale computational docking for anti-cancer drugs.

## 1. Introduction

Cancer is one of the leading causes of death in the US and worldwide [1,2]. It is a source of significant health-related suffering that places an outstanding economic burden on society [3]. Just in 2019, the projected patient out-of-pocket cost for cancer treatment in the US was $16.22 billion [4]. Thus, cancer treatment is a focal point of multiple high-profile health initiatives, national, e.g., the 21st Century Cures Act by the US Congress, and global, e.g., The Global Breast Cancer Initiative by WHO [5,6,7,8]. Such initiatives facilitate data generation and sharing between research groups from different scientific fields, assisting the development of novel treatments. They help advance disease prevention, early diagnostics, and treatment development which are all non-trivial tasks. Multiple data modalities are used to elucidate cancer mechanisms—clinical records, genetic sequences, transcriptional expression, and cytological imaging [9,10,11,12,13,14].

It is well known that cancer is a set of complex genetic disorders that can manifest with significant differences between patients [15,16,17]. Tumors of the same histology type can respond differently to a treatment [18,19,20]. Thus, drug response prediction is of paramount importance for designing personalized cancer treatment. The anti-cancer drug response prediction problem is defined as follows—given cancer representations and drug representations, predict a treatment efficacy. Cancers are usually represented by their genomic/molecular or phenotypic profiles, such as transcriptomics, mutations, DNA methylations, pathology images, and others [21,22,23,24]. Drug representations can come from multiple sources—such as molecular descriptors and fingerprints, SMILES, and graphical representations [24]. In in vitro drug screening experiments, the treatment response is usually summarized based on the dose-response curves fitted to the cell viability readouts obtained at multiple drug concentrations. Some commonly used response metrics include the area under the dose-response curve (AUC), the half-maximum inhibitory concentration (IC50), and others [25]. In in-vivo drug screening experiments, treatment responses can be measured by metrics like tumor volume change over time [26]. The current work focuses on pre-clinical drug response studies conducted primarily in immortalized cell lines. While state-of-the-art experimental techniques like in vitro profiling utilizing 3D organoids or in vivo profiling using patient-derived xenografts (PDX) models can provide more accurate insights for clinical trials [27], cell line drug response studies remain a versatile instrument for initial drug screening. It is important to recognize that cell lines cannot perfectly model biological processes in vivo, and to maximize the efficiency of solving real-world problems such as precision medicine we need to employ more comprehensive data integration strategies such as a virtual molecular tumor board [28]. However, the limited availability of this data prevents us from constructing ML models directly from them. The common approach for the pre-clinical drug response models is to utilize transfer learning from cell lines.

Researchers have approached the problem of anti-cancer drug response prediction via diverse methodologies. These include traditional machine learning (ML) algorithms, such as support vector machine (SVM) [29,30], random forest (RF) [31,32,33], and boosting algorithms (e.g., AdaBoost, XGBoost, and Light Gradient Boosting Machine—LightGBM) [34,35,36,37]. Recently, an emerging trend has been to develop and apply various deep learning (DL) architectures for drug response prediction [38]. Fully connected deep neural networks have been used to predict IC50 from in vitro drug screening experiments [39]. Convolutional neural networks (CNN) are used by the DeepIC50 and DeepCDR models to integrate drug features and cell line molecular data [40,41] for response prediction. REFINED and IGTD convert tabular molecular data of cell lines and drugs into images, to leverage the strong capability of CNN architectures in exploiting spatial relationships between features for making predictions [42,43]. There are also several autoencoder-based models, adversarial networks, Bayesian neural networks, collaborative filtering, and graph neural networks (GNN)-based approaches used for drug response prediction applications [44,45,46,47,48,49]. The attention mechanism is used in a few recent approaches, such as PaccMann, CADRE, GraTransDRP, and DeepTTA [24,44,50,51,52]. These models predominantly use transformer-based modules to create drug embeddings either directly from SMILES or other representations, e.g., explainable substructure partition fingerprints (ESPF) [53,54]. In terms of task formulation, existing drug response prediction methods take two major routes. Some of them discretize response values into ‘responsive’ and ‘non-responsive’ categories and perform classification analyses, while others directly perform regression analyses on the continuous treatment response metrics, such as IC50 and AUC.

Most existing anti-cancer drug response models make predictions based on representations of cancers and drugs. Models built on these cancer and drug representations are expected to integrate their information and extract features related to the treatment mechanism for making predictions. However, despite the extensive exploration of various modeling approaches and feature representations, anti-cancer drug response prediction remains a challenging task [38,55], without a standard approach that can be routinely used in actual drug development and clinical practice. A reasonable conjecture on the potential reason for the difficulty of modeling drug response based on cancer and drug features is that the current data and modeling approach might not sufficiently characterize the complex interactions between molecular cancer systems and drug molecules for modeling response mechanisms.

To meet this challenge, we investigate a new category of features that should elucidate the molecular mechanisms of action (MMoA) and explicitly characterize the cancer-drug interactions to assist response modeling—large-scale computational docking scores for protein-ligand complexes [56,57]. These MMoA-related features are expected to bridge the gap between cancer and drug representations, and thus help the prediction models to integrate their features for better modeling of response mechanisms. This work is a proof-of-concept study and is not intended to explore either a comprehensive list of potential MMoA features or all potential ways of integrating them into prediction models, though, we describe some of them in this paper. We focus on incorporating one of the most common methods for virtual drug screening—molecular docking—into the feature generation process as a proxy for ligand-protein interaction. We construct a blind docking pipeline using the OpenEye suite [58] to estimate the binding propensities between drug molecules and proteins targeted by anti-cancer drugs approved by the U.S. Food and Drug Administration (FDA). The computationally derived binding scores are used as features in addition to the cancer and drug representations for predicting drug response [24,58]. Docking scores serve as a proxy for potential alternative protein-ligand binding propensity. It is natural to incorporate structure-based information on potential interactions between ligands and proteins. However, our studies indicate that they contain a limited amount of information relevant to drug response on top of existing chemical descriptors.

This work has several unique contributions to the research on anti-cancer drug response modeling. First, to our knowledge, our study is the first analysis of incorporating structure-based MMoA features into drug response modeling that directly links drug properties with the cancer molecular system via molecular docking. We are investigating whether the addition of MMoA features, such as binding affinity estimates, will improve the performance of drug response modeling. Second, we estimate the binding affinities between protein targets of FDA-approved anti-cancer drugs and compounds included in several major cell line drug screening datasets and provide the obtained binding scores as a public resource for the research community. These binding affinity estimates can be used for other drug discovery studies, such as drug target identification and drug response modeling on other types of cancer models. Third, our results demonstrate that the integration of binding scores into response modeling is beneficial and shall be considered by future research.

In this paper, we argue that the introduction of novel molecular mechanism of action (MMoA) features can help to bridge the gap between different data modalities and improve the performance of the ML models for cancer drug response prediction. Additional information should allow non-linear models to enhance the saliency of the feature combination process.

## 2. Materials and Methods

### 2.1. General Outline

**Figure 1:**
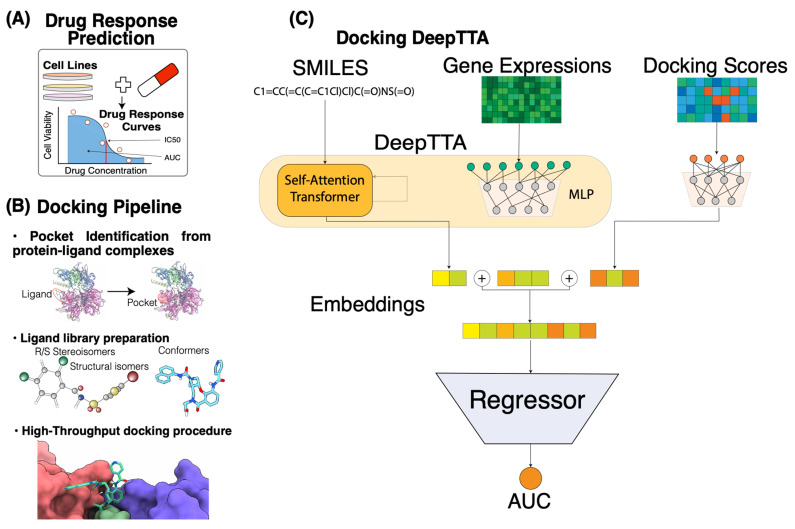
Overview of docking scores integration in drug response prediction pipeline. (**A**) The setting of the drug response prediction problem in our study. The central entity here is a drug response curve that reflects the cell viability at different drug concentrations. In this study, we focused on predicting the AUC response value using gene expressions and compound information. (**B**) Blind docking pipeline. Bullet point steps (from top to bottom): creating receptors from the existing protein-ligand complexes using Spruce; ligand library preparation using OpenEye suite tools Flipper (stereocenters enumeration for R/S and *cis*/*trans* stereochemistry), Tautomers (enumeration and canonicalization of tautomeric forms), and OMEGA (conformer generator); rigid docking using FRED. (**C**) Machine learning pipeline based on DeepTTA algorithm. Input consists of drug SMILES representations and cell line gene expressions that are fed to the self-attention transformer and multi-layer perceptron (MLP) components, respectively. Docking score embeddings are generated using a separate MLP component.

We use high-throughput molecular docking to build interaction profiles for drug molecules and protein targets of FDA-approved anti-cancer drugs. The idea behind this is to highlight the underlying mechanism of actions (MoAs) of drugs by estimating their binding affinities with known protein targets of anti-cancer drugs. The usage of continuous measures like docking scores instead of binary ones for a protein-ligand interaction helps to integrate information about the physical properties of small molecules and target proteins in a more refined manner, as it not only indicates interaction preferences but also provides estimates on their degrees. In this study, we consider 1262 drugs and 2093 distinct structures of protein-ligand complexes from the Protein Data Bank (PDB) database. For the docking analysis, we conducted ligand library preparation and developed a high-throughput docking protocol using toolkits in the OpenEye program suite. After generating the binding scores, we built and evaluated response prediction models based on cancer cell line drug screening datasets. Cell line gene expressions and drug molecular descriptors or Simplified Molecular Input Line Entry System (SMILES) strings were used as primary input features of the models. We compared the prediction performances of models with and without the binding scores as additional input features. Three different model algorithms were used to build the drug response prediction models, including LightGBM [59], a fully connected neural network (FCNN) [60], and DeepTTA [24]. The outline of drug response problem and our approach is described in Figure 1.

### 2.2. Cell Line, Drug, and Response Data

Our data used for analysis consists of five parts—gene expression profiles of cell lines, drug SMILES strings, drug molecular descriptors, GaussChem4 docking scores, and drug response measurements. We use two drug response datasets for analysis, which are the Cancer Cell Line Encyclopedia (CCLE) [61] and the Cancer Therapeutics Response Portal (CTRP) [62] datasets. The CCLE dataset includes cell viability measurements of 8950 experiments conducted with 24 compounds and 474 cell lines. The CTRP dataset includes 254,566 experiments involving 495 compounds and 812 cell lines. Quality control for the CCLE dataset was performed by verifying concordance between genotypes detected by sequencing and SNP arrays to ensure that there were no mix-ups between samples, and sequencing reads aggregated from different barcoded pools were checked for genotype concordance, to ensure sample identity. The CTRP utilizes publicly available gene expression annotations for cancer cell lines, effectively unifying most stand-alone quality-controlled small datasets from NCBI by conducting drug response experiments in standardized conditions.

To obtain the drug response value of each experiment, we fitted a hill-slope model to viability readouts at multiple doses to draw the dose-response curve. Afterward, we calculated the area under the curve (AUC) for the dose range of [10^−10^ M, 10^−4^ M], which was then normalized by the length of the dose range. After normalization, the AUC value takes a range from 0 (complete response) to 1 (no response). The fixed-dose range from 10^−10^ M to 10^−4^ M for calculating AUC values ensures the integral characteristic of AUC values for comparisons between experiments that were originally conducted across different dose ranges.

For the gene expression profiles of cell lines, we include a set of “landmark” genes [63] derived from the Library of Integrated Network-Based Cellular Signatures (LINCS) [36] project as well as oncology-associated genes from OncoKB [64] and Genomics of Drug Sensitivity in Cancer (GDSC) [65]. We also make sure that all genes associated with the protein complexes for which binding scores were computed are included in the expression profile. The expression data were retrieved from the CCLE resource and TPM (Transcripts Per Million reads mapped) values were used as expression values. In total, the expression profile of cell lines includes 2019 genes. Gene expressions were standardized using the Z-transformation so that each gene has a zero mean and a unitary standard deviation across cell lines. Scaler parameters were computed based on the training set during each cross-validation step and then applied to the validation and testing sets. This was done to reduce information leakage between training and evaluation data partitions. Docking scores and drug molecular descriptors were also processed via the same protocol so that these drug features were standardized across drugs based on the training set.

Two different drug representations, SMILES strings, and molecular descriptors, were used in the analysis. FCNN and LightGBM accept drug descriptors as input drug features, while DeepTTA infers drug features from SMILES strings. More details on the data transformations performed by DeepTTA are available in the section that introduces the DeepTTA method and in the original publication [24]. The Dragon v.7.0 software package [66] was used to compute 1623 numerical molecular descriptors for the drugs. The MMoA features of drugs are Chemgauss4 scores obtained using the OpenEye software suite OEDocking 4.2.1.1 [67]. These scores incorporate Gaussian smoothed potentials that estimate the complementarity of ligand poses to a protein pocket based on metal–chilator interactions, shape, and hydrogen bonding interaction between ligand, protein, and solvent. A lower score corresponds to a better fit.

### 2.3. Curation of PDB Structures of Anti-Cancer Drug Target Proteins

We generated a list of Protein Data Bank (PDB) structures of anti-cancer drug target proteins via two steps [68]. First, we collected information on FDA-approved anticancer drugs (including their drug target genes) from CenterWatch (https://www.centerwatch.com/ (accessed on 30 June 2019). CenterWatch is a recognized global leader in providing clinical trial information. Second, we used a mapping between gene Entrez IDs and associated PDB protein structure IDs from UniProt [69,70] to identify the PDB IDs associated with the drug target genes. We included only proteins with resolved protein–ligand complex structures documented by the PDB database. Finally, we obtained 2093 PDB structures of protein–ligand complexes in which proteins correspond to 155 unique genes.

### 2.4. Creation of Receptors from Existing Protein-Ligand Complexes

A pocket search was conducted using Spruce (Version 1.5.0.1) with parameters *allow_validation_error* and a maximum number of atoms equal to 300 in the system. Spruce splits existing protein-ligand complex and isolates active sites where small molecules are bound to macromolecules [71]. Running Spruce allows us to make necessary preparations to improve the quality of protein structures. It performs multiple tasks, including modeling missing loops, filling in missing pieces for chain breaks and partial sidechains, fixing protein backbone atoms and incorrect covalent bonds to metals, adding hydrogen atoms, and optimizing their placement. Spruce expands asymmetric units to its biological counterpart for the X-ray crystallography structures. Spruce successfully created OpenEye design units for the 2093 protein–ligand complexes. Afterward, we used the ReceptorInDU utility from OEDocking 4.2.1.1 to set up docking-ready receptors from the obtained design units.

### 2.5. Preparation of Compound Ligand Library

We used the OMEGA [72,73] and QUACPAC [74] toolkits from the OpenEye suite to prepare a set of 3D ligand structures for drugs using their SMILES strings. First, we used the flipper [72] (Version 4.2.0.1) program to enumerate stereocenters of the molecules—R/S stereochemistry and cis/trans stereoisomers. This program determines atomic stereocenters based on graph algorithms. Second, we ran Tautomers (Version 2.2.0.1) to produce the most probable structural isomers expected to be present in the aqueous phase. Third, we used OMEGA (Version 4.2.0.1) to generate conformers for the given isomers. OMEGA reviewed multiple ring conformations and invertible nitrogen atoms to identify plausible 3D models. We recorded 100 distinct conformations for each compound for the subsequent docking analysis.

### 2.6. High-Throughput Docking Procedure

We performed computational experiments for virtual screening using the FRED (Version 4.1.2.1) molecular docking software [75,76]. It runs an exhaustive search of possible positions of a given ligand with different rotations and translations within a receptor site. Both protein and ligand remain rigid during the docking process. FRED uses the Gausschem4 score to estimate the fitness of a pose [58]. The Gausschem4 score determines the complementarity between the receptor site and a drug molecule based on Gaussian smoothed potentials. It considers the shape, hydrogen bonds between a small molecule and a protein, interactions with implicit solvents, and metal-chelator interactions. To facilitate computing for docking, we utilized FRED with Message Passing Interface (MPI) parallelizations. We split the workload across 128 CPU cores on a computer server. It took 124 h to complete the docking analysis with a total workload of 15,872 CPU hours, generating a matrix of the GaussChem4 scores with the 2093 rows corresponding to PDB structures and 1262 columns corresponding to drugs. For each combination of a PDB structure and a small molecule, the best docking score was recorded. The missing rate in the data matrix is 4.9%, resulting from implausible initial structures, e.g., positioning comparatively large drug molecules in a small pocket.

### 2.7. LightGBM, FCNN, and DeepTTA Models for Drug Response Prediction

LightGBM is an efficient implementation of the gradient boosting decision tree (GBDT) model [59]. A distinctive feature of LightGBM is the incorporation of two heuristics that respectively reduce the number of samples and features used for a single boosting step—Gradient-based One-Side Sampling (GOSS) and Exclusive Feature Bundling (EFB). These heuristics efficiently reduce computational workload and allow for training lightweight but efficient models. GOSS identifies under-trained data points, which are samples with the largest gradients that significantly contribute to information gain. It allows maximizing an information gain for each boosting step while limiting the portion of the data set used for the construction of a decision tree and, thus, computational complexity. EFB bundles together mutually exclusive features, e.g., one-hot encodings via a graph coloring problem approximation. This allows the program to reduce the number of features it must consider. We use an ensemble of regression trees implemented in the LightGBM package as one of the models for cancer drug response prediction. We used 2000 maximum boosting steps in the model with early stopping based on the validation set and 100 early stopping rounds. The loss function for model training is a mean square error (MSE). Gene expression profiles and drug descriptor profiles are concatenated for input into the LightGBM model. The binding scores with all considered receptors are also concatenated with gene expressions and drug descriptors when they are used as input features for response prediction.

Fully Connected Neural Network (FCNN) in our study has a standard Multi-Layer Perceptron (MLP) architecture where each output of the previous layer is connected to all inputs of the next one. This neural network takes concatenated vectors of cell line gene expression profiles and drug molecular descriptors as the input. The FCNN has 10 hidden dense layers of sizes 4096, 2048, 1536, 1024, 768, 512, 256, 128, 64, and 32 and the output layer has a single output. The activation function in each layer is a rectified linear unit (ReLU). The network was trained with a batch size of 512 for 100 epochs. The loss function for model training was MSE. The Adam optimizer was used for optimization with a learning rate of $10^{-4}$. The number of training epochs was fixed, but to avoid overfitting, we picked the model with the highest performance (lowest MSE) on the validation set.

DeepTTA is a recently developed model for drug response prediction that exhibits a competitive prediction performance [24]. It has a hybrid structure, with two separate modules for representation learning of cell line gene expressions and drug SMILE strings. The module that encodes gene expressions is an MLP consisting of three hidden layers with dimensionalities of 1024, 256, and 64. The drug representation learning module first converts drug SMILES strings into Explainable Substructure Partition Fingerprints (ESPF) derived from ~2 million compounds to encode ~2700 molecular substructures [54]. Then, a transformer encoder is built to capture contextual information from the drug substructures and uses an attention mechanism to derive drug representations. To unify the input format across multiple drugs, DeepTTA uses the following approach. It defines a substructure vocabulary D over the entire drug corpus, then generates a substructural sequence $S=\{S_{1},S_{2},\ldots ,S_{l}\;\}$ for each drug, where l is the number of the drug substructures and $S_{i}$ is an individual substructure token. Then, an intermediate representation of each drug denoted by $e_{i}$ is calculated based on ESPF values. It is a sum of content representation $C_{i}=W_{c}M_{i}^{s}$ and positional representation $P_{i}=W_{pos}I_{i}$. Content representation reflects the abundance of the substructures in the small molecule. It is adjusted via a learnable dictionary lookup matrix $W_{c}$. $M_{i}^{s}$ is the i-th row in the matrix of one-hot encoded substructures for all drugs, corresponding to the i-th drug. Positional representation captures positional information of the drug substructures. It is encoded by a one-hot vector $I_{i}$ that has the i-th position equal to 1, and a lookup dictionary $W_{pos}$. The representation $e_{i}=C_{i}+P_{i}$ is then transformed by the multi-attention layer [77]:

$$Attention\left(e_{i}\right)=softmax\left(\frac{\left(e_{i}W^{q})(e_{i}W^{k}\right)}{\sqrt{d}}\right)\times \left(e_{i}W^{v}\right),$$

where $W^{q}$, $W^{k}$, and $W^{v}$ are learnable weights and $\frac{1}{\sqrt{d}}$ is a scaling factor. The embedding outputs from the two representation learning modules of gene expressions and drugs are concatenated and then forwarded to an MLP for drug response prediction. When adding docking scores as additional features for response modeling, the architecture of DeepTTA is modified. A separate MLP module is devised to encode drug docking scores into embeddings. It includes three hidden layers with sizes of 512, 128, and 32. The docking score embeddings are concatenated with the embeddings of drugs and gene expressions. The concatenated embeddings are forwarded to an MLP with hidden layers of the sizes of 1024, 1024, and 512 for making response predictions. When training DeepTTA with and without docking scores, we used the Adam optimizer with a learning rate of 0.0001, 100 epochs, batch size of 512, and a dropout rate of 0.1. To avoid overfitting, we saved only the model with the highest performance on the validation set.

### 2.8. Performance Evaluation Scheme

All three models, including LightGBM, FCNN, and DeepTTA, were trained and evaluated through 10-fold cross-validation (CV). During each CV iteration, 80% of the data was designated to the training dataset, 10% to the validation set, and 10% to the test set. All three models used the same data partitions for cross-validation analysis for a fair comparison. The metrics used for evaluating prediction performance include the coefficient of determination (R^2^), pearson correlation coefficient (PCC), and spearman correlation coefficient (SCC). A detailed description of the performance metrics is available in Appendix A. To evaluate the usefulness of docking scores for drug response prediction, we train and assess the three prediction models with and without docking scores as input features. The prediction performance obtained using gene expressions and drug descriptors/SMILES was compared with that obtained using binding scores in combination with gene expressions and drug descriptors/SMILES. The paired t-test was conducted to evaluate the statistical significance of the performance difference, and the Benjamini-Hochberg procedure was applied for multiple test corrections to control the false discovery rate (FDR) [78].

## 3. Results

**Figure 2:**
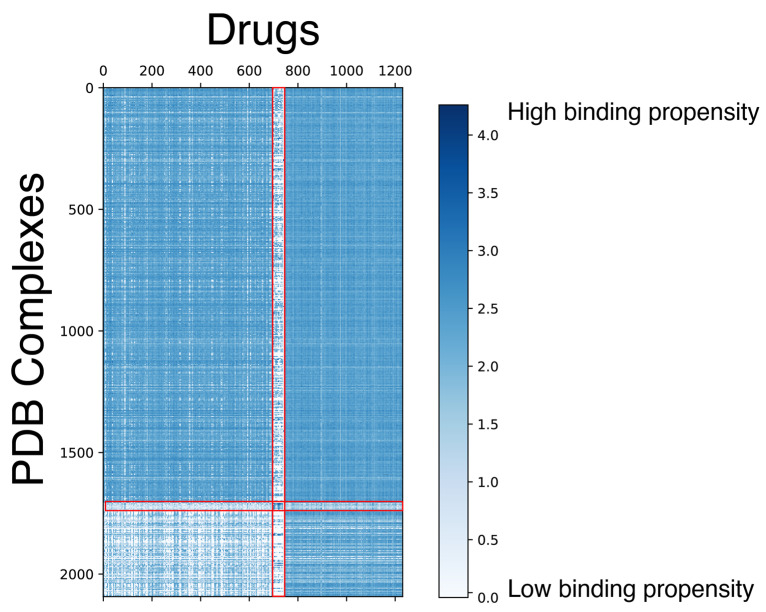
Spectral co-clustering of docking scores. The result shows patterns of biclusters (i.e., clusters representing a relatively homogeneous subset of both PDB structures and drugs). The red lines highlight the visible borders of the data substructures with the different levels of high-scoring drug-PDB complex pairs sparsity. Each score corresponds to the pair of a PDB structure (*Y*-axis) and a small molecule (*X*-axis). The docking score is capped at 0 for visualization purposes to provide a better view of the protein-drug pairs with high binding chances.

After obtaining the docking scores, we performed a clustering analysis on the docking score matrix using spectral co-clustering [79] (Figure 2). This analysis was done to find patterns in GaussChem4 binding scores produced by FRED docking software (Version 4.1.2.1) for pairs of drugs (*X*-axis) and active binding sites in anti-cancer drug target proteins (*Y-axis*). The data matrix contains information for all combinations of 1262 drugs and 2093 active binding sites (protein receptors). We considered 100 distinct conformers for each small molecule, and only the highest pose score was recorded. In the original data matrix, a high GaussChem4 score represents an unlikely interaction. The low scores represent highly likely interactions. For visualization purposes, we apply the following transformation for every GaussChem4 score:

$$\log\left(x_{max}-x_{i}+1\right)$$

where $x_{i}$ is the GaussChem4 score being transformed and $x_{max}$ is the maximum value in the original score matrix. After transformation, high values indicate highly likely interactions, while low values indicate unlikely interactions. When we apply spectral co-clustering to the transformed binding data, we observe a small group of “clean” drugs—compounds with high selectivity [80]—that do not interact with most of the PDB structures denoted by the thin white vertical line. There is also a group of cancer targets that are challenging for most explored drugs to pick up (bottom left square). As the gausschem4 score used by OpenEye software (Version 4.1.2.1) is not directly comparable between different binding pockets and we caution our readers and dataset users from concluding cross-target comparison.

**Figure 3:**
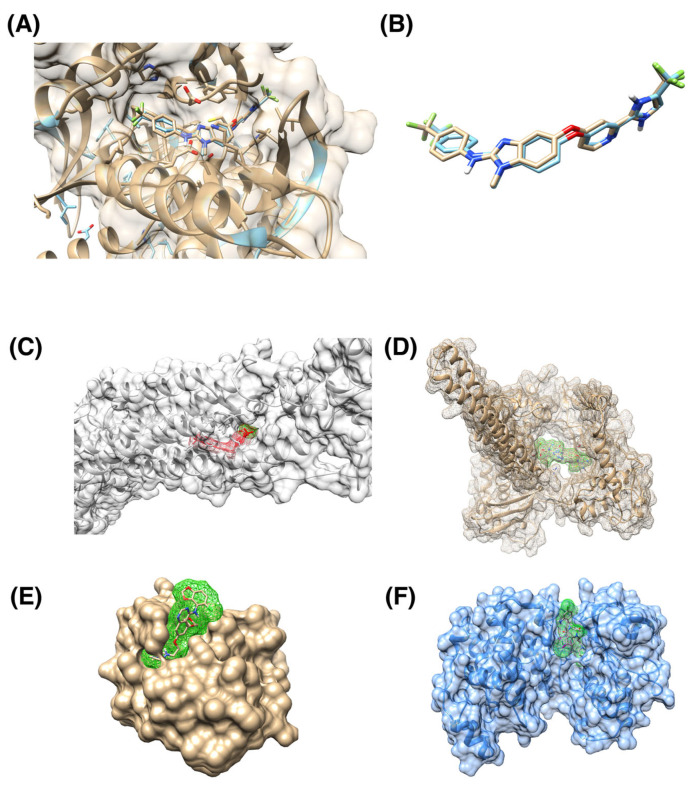
Examples of docking ligands into various protein targets. (**A**) Superposition of reference 5CT7 PDB structure of BRAF in complex with RAF265 drug (cyan) with RAF265 docking posture into the same protein (brown). (**B**) Superposition of reference RAF265 posture (cyan) and docked posture (brown). The ligands are the same as in panel (**A**), with the protein structure removed to provide a clearer view. RMSD between two ligand postures is 0.751 Å, indicating a good consistency between the two postures; the corresponding binding score is −20.06. (**C**) Human smoothened receptor complex (grey) with docked RAF265 (red); the corresponding binding score is −18.91. (**D**) Human DNA Topoisomerase (brown) with docked Targegen B-Raf/PDGFR inhibitor Cpd 6 (highlighted with green); corresponding docking score is −15.59. (**E**) Ubiquitin binding pocket of the HDAC6 zinc-finger domain (brown) with docked saracatinib (highlighted with green); corresponding docking score is −2.47. (**F**) p38 MAPK (blue) with docked JNJ-27291199 compound (highlighted by green). The gausschem4 score is 15.02.

We included a case study for the RAF265 drug to validate our docking procedures. We calculate the root mean squared distance (RMSD) between ligands in reference PDB structure and RAF265 posture from our blind docking protocol (Figure 3A,B). The resulting RMSD is 0.751 Å, which indicates good docking quality. We also include examples of ligands being docked in pockets that differ from the corresponding reference PDB structure (Figure 3C–F).

**Figure 4:**
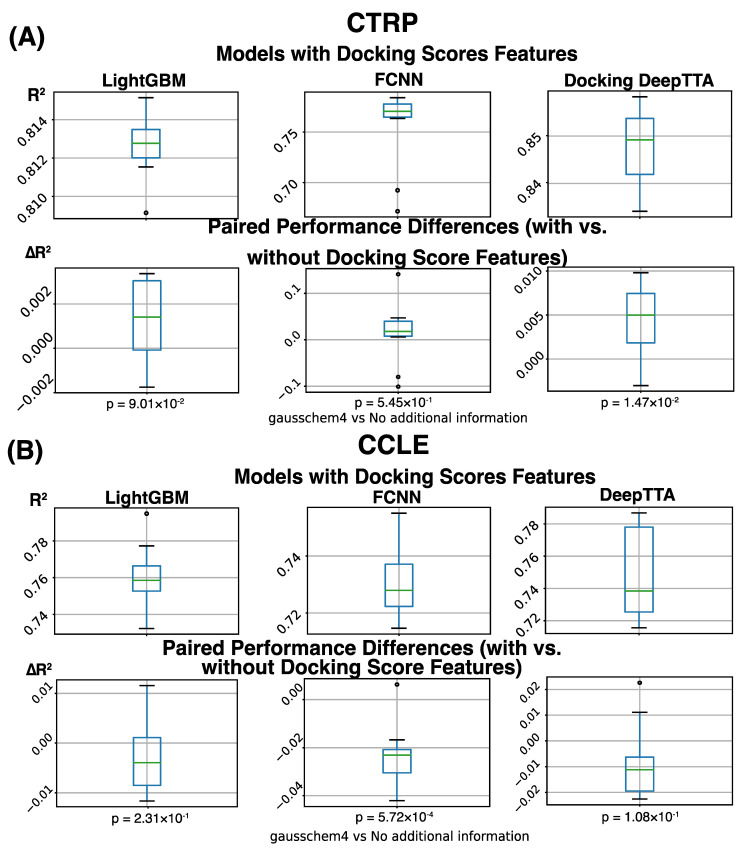
Drug response prediction performance (R^2^) was obtained for different models, and data sets, and a difference in performance for models with and without incorporating molecular docking information. Boxplot represents four data distribution quantiles, with the green line representing mean. (**A**) Overview of prediction performance obtained on the CTRP dataset. The first row of plots shows that the average $R^{2}$ of the models with binding score features is 0.813 for LightGBM, 0.755 for FCNN, and 0.848 for DeepTTA. The second row of plots shows the performance difference between models with and without binding score features, which is calculated for every CV run. The average performance difference of LightGBM, FCNN, and DeepTTA is 0.013 (*p* = 0.55), 0.0133 (*p* = 0.09), and 0.0045 (*p* = 0.015), respectively. (**B**) Overview of prediction performance obtained on the CCLE dataset. The first row of plots shows the average prediction performance of models with binding score features, which is 0.764 for LightGBM, 0.730 for FCNN, and 0.749 for DeepTTA. The second row of plots shows the performance difference caused by adding binding score features.

The models we assess in this work are LightGBM, FCCN, and DeepTTA. We evaluate these models’ drug response prediction performance on the CCLE and CTRP datasets. Particularly, we also investigate the effect of adding docking score features for response modeling. To do this, we calculate performance metrics for the models without docking score features (Table 1) and compare them with the results from the models trained on data with expanded drug information incorporating docking scores (Table 2). Overall, we observe that the performance difference resulting from adding docking score features is marginal (Figure 4A). Measured by R^2^, the average performance difference obtained through cross-validation is in the range of [−0.0231, 0.0133] for the six performance comparisons across three models and two datasets. Two out of the six comparisons show a statistically significant difference (adjusted *p*-value ≤ 0.05).

On the large CTRP dataset, we see a consistent benefit of adding docking scores for response modeling (Table 3). The performance difference caused by adding docking scores is always positive for all combinations of models and metrics on the CTRP dataset, which indicates their beneficial impact on response modeling (Table 3). Measured by the SCC, the performance improvement is consistently statistically significant (adjusted *p*-value ≤ 0.05), showing that the order of response values is always better predicted on the CTRP dataset when binding scores are used. The state-of-the-art drug response prediction model, DeepTTA, also shows a statistically significant improvement in prediction performance measured by all three metrics (Table 3).

On the small CCLE dataset, we do not observe a consistent improvement in prediction performance when adding binding scores (Table 3), probably due to limited drug diversity and model over-fitting. Compared with the CTRP dataset, the CCLE dataset includes much fewer drugs, which may limit the power of binding scores as additional drug features for response modeling. On a small dataset like CCLE, adding more features for prediction can lead to model over-fitting, especially for models like FCCN with a massive number of parameters and a deep architecture. The small validation set used in cross-validation may need more diversified drug and cell line combinations to prevent over-fitting. Table 3 shows that when adding binding scores, the prediction performance of FCCN is statistically significantly decreased (adjusted *p*-value ≤ 0.05) measured by all three performance metrics. Besides FCNN, adding docking scores to the other two models trained and tested on the CCLE dataset does not have a statistically significant effect (Figure 4).

## 4. Discussion

State-of-the-art deep learning models in the drug response prediction field rely on the following general architecture. Genetic information and drug descriptors are encoded by separate network submodules. Then the obtained representations of biological samples and compounds are fed together into the discriminator part of the neural network. On the one hand, it allows researchers to manage model complexity efficiently. Such an approach makes it possible to fine-tune source-dependent parts of the model or make use of transfer learning approaches to produce numerical representation for each interacting data modality. On the other hand, it limits the non-linear combinations of the input features between different modalities.

Biomedical research produced large arrays of multimodal data (e.g., gene expression, cytology imaging, methylation) that could be integrated to elucidate the MMoA effects of drugs on the in vitro biological models and, ultimately, on human organisms. However, cancer can introduce significant changes to the way biological processes behave. These changes can be due to the disruptive effects of cancer mutations and aberrant alternative splicing. So, a natural extension of our analysis is the system-level study of protein-protein interaction (PPI) network rewiring [81,82,83].

The current docking pipeline has several limitations. First, in our study, we used only existing experimentally validated protein receptors for calculating binding scores. It provides credible estimates for pockets on a specific protein. However, this approach is naïve as it does not necessarily provide a comprehensive picture of the potential drug effect on a given protein. If a pocket is bound by a drug, it is not necessarily a good target for another drug to bind. Instead, an alternative active site of the protein may be responsible for the potential interaction with the second drug. Moreover, most experimentally validated PDB structures are mostly for wild-type proteins; cancer-related structural variations, such as genetic mutations and their allosteric effects or aberrant alternative splicing, which can alter the receptors, are not considered. This issue can potentially be addressed by expanding and using more accurate receptors provided by either receptor search methods or existing databases. The Spruce toolkit used in the analysis does not identify novel protein binding sites. Another OpenEye toolkit, SiteHopper, can perform a rapid search to identify potential protein receptors [84]. OpenEye also provides a separate receptor database generated by SiteHopper and Spruce. Different versions of this database cover ~40,000 and ~300,000 potential binding sites. Second, the current protocol uses rigid docking, limiting modeling precision for flexible regions. With the increasing variety of potential binding sites and drug molecules that do not necessarily conform to Lipinski’s Rule of five (Ro5) [85,86,87], traditional docking methods can miss potential interactions. An alternative, more informative, and accurate approach is molecular dynamics (MD) simulation. However, conducting the computationally heavy MD simulations for >2,000,000 pairs of drug molecules and protein receptors is computationally prohibitive, so we opted out of doing it. Third, certain classes of molecules are also excluded from the analysis, e.g., alternative splicing modulators [88,89], as there were no available combinations of PDB structure and drug response studies found.

The current study focused on utilizing a traditional docking approach to gain additional information on drug activity across a wide range of cancer targets. We see an opportunity to obtain more comprehensive binding score profiles for the drugs in extending the analysis to a larger number of receptors. This analysis requires a much higher computational efficiency that common docking programs cannot provide, necessitating the usage of a surrogate model. Current surrogate docking models primarily focus on screening large numbers of compounds for a few carefully curated binding pockets [90,91,92], which prompts a need to construct a comprehensive binding score prediction model that can predict bindings across multiple protein targets based on inputs of both compound and protein target. We envision the development of such a highly efficient surrogate model as our next step.

## 5. Conclusions

Existing drug response prediction methods lack meaningful incorporations of the physical properties of ligands and proteins, especially their interactions [24,93]. We demonstrate that incorporating features generated through computational docking enhances the performance of state-of-the-art drug response prediction models without adversely affecting the predictions of simpler models. Our analysis results show a more robust performance improvement on large drug screening data with more diversified drugs. The SCC performance measurements of all three prediction models are statistically significantly improved on the large dataset after adding docking score features, indicating that the true responses and the predicted responses become more consistent in terms of ranking relationship. However, we also highlight the limitations of this approach and note that along with the usage of highly informative drug features derived from molecular descriptors or SMILES strings, the observed performance impact on the drug response prediction models is marginal. 

## Figures and Tables

**Table 1:** The baseline performance of the three explored models on the CCLE and CTRP datasets with no binding score information incorporated. The table reports the mean and standard deviation of performance metrics based on ten cross-validation runs.

Docking Information		Not Used					
Dataset		CCLE			CTRP		
	Metric	R^2^	PCC	SCC	R^2^	PCC	SCC
Method							
FCNN		0.753 ± 0.009	0.869 ± 0.005	0.768 ± 0.008	0.742 ± 0.040	0.864 ± 0.023	0.839 ± 0.006
LightGBM		0.764 ± 0.019	0.874 ± 0.011	0.791 ± 0.018	0.811 ± 0.001	0.901 ± 0.001	0.852 ± 0.001
DeepTTA		0.758 ± 0.022	0.873 ± 0.012	0.779 ± 0.018	0.843 ± 0.007	0.919 ± 0.004	0.878 ± 0.008

**Table 2:** The performance of the three models on the CCLE and CTRP datasets with the GaussChem4 docking scores as additional features. The table reports the mean and standard deviation for each performance metric calculated based on ten cross-validation runs. Bold text indicates experiments in which the addition of binding affinity information increases the mean of the performance metrics.

Docking Information		GaussChem4 Scores					
Dataset		CCLE			CTRP		
	Metric	R^2^	PCC	SCC	R^2^	PCC	SCC
Method							
FCNN		0.730 ± 0.012	0.856 ± 0.007	0.749 ± 0.014	**0.755 ± 0.039**	**0.871 ± 0.022**	**0.847 ± 0.003**
LightGBM		0.761 ± 0.017	0.873 ± 0.010	0.788 ± 0.016	**0.813 ± 0.002**	**0.902 ± 0.001**	**0.853 ± 0.002**
DeepTTA		0.749 ± 0.028	0.873 ± 0.014	**0.781 ± 0.022**	**0.848 ± 0.008**	**0.921 ± 0.004**	**0.883 ± 0.007**

**Table 3:** The performance difference between models with and without binding score features averaged across CV runs. The performance difference is calculated for every CV partition (i.e., training, testing, and validation sets) based on models trained and evaluated using the CV partition. A positive difference indicates the beneficial influence of binding score features. The *p*-values are obtained from paired *t*-tests across CV runs and corrected by the BH procedure. Bold text indicates statistically significant performance differences (adjusted *p*-value ≤ 0.05).

Docking Type		Differences					
Dataset		CCLE			CTRP		
	Metric	R^2^	PCC	SCC	R^2^	PCC	SCC
Method							
FCNN		**−0.0231** **(*p* = 5.72 × 10^−4^)**	**−0.0133** **(*p* = 5.20 × 10^−4^)**	**−0.0191** **(*p* = 1.21 × 10^−4^)**	0.0133 (*p* = 5.45 × 10^−1^)	0.0073 (*p* = 5.54 × 10^−1^)	**0.0077** **(*p* = 9.26 × 10^−3^)**
LightGBM		−0.0029 (*p* = 2.31 × 10^−1^)	−0.0016 (*p* = 2.38 × 10^−1^)	−0.0036 (*p* = 1.52 × 10^−1^)	0.0013 (*p* = 9.01 × 10^−2^)	0.0007 (*p* = 9.51 × 10^−2^)	**0.0013** **(*p* = 8.38 × 10^−3^)**
DeepTTC		−0.0084 (*p* = 1.08 × 10^−1^)	0.0001 (*p* = 9.52 × 10^−1^)	0.0017 (*p* = 5.23 × 10^−1^)	**0.0045** **(*p* = 1.47 × 10^−2^**	**0.0024** **(*p* = 1.87 × 10^−2^)**	**0.0048** **(*p* = 1.69 × 10^−2^)**

**Figure A1:**
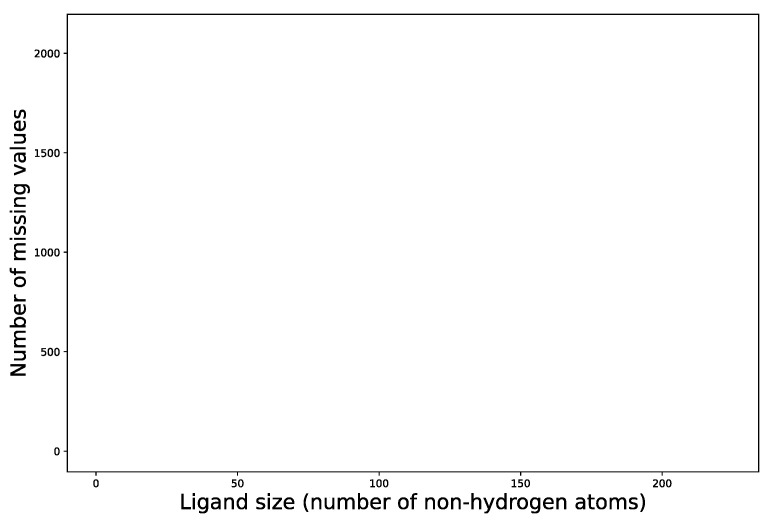
Plot of the number of missing data points per ligand based on the ligand size. Large ligands contribute to the majority of missing data.

## Data Availability

Data generated by this study will be available in Supplementary Materials. Code is available over the reference: https://github.com/AlexandrNP/BindingScoresDRP (accessed on 1 November 2023).

## Supplementary Materials

The following supporting information can be downloaded at: https://www.mdpi.com/article/10.3390/cancers16010050/s1, Table S1: gausschem4 scores matrix for drugs and PDB structures; Table S2: Drug identifiers map.

## Appendix A

### Appendix A.1. Performance Metrics

#### Appendix A.1.1. Coefficient of Determination $R^{2}$

The coefficient of determination indicates the variance proportion of the variable of interest that is explained by a given regression model.

$$R^{2}=1-\frac{{SS}_{res}}{{SS}_{tot}},\;\;{SS}_{res}=\sum_{i=1}^{n}{\left(y_{i}-f_{i}\right)}^{2},\;\;{SS}_{tot}=\sum_{i=1}^{n}{\left(y_{i}-\bar{y}\right)}^{2},$$

where ${SS}_{res}$ is a residual variance from the regression model, ${SS}_{tot}$ is a total variance, n is a total number of points in the evaluation set, $y_{i}$ is the true value of the i-th point, $f_{i}$ is the predicted value of the i-th point, $\bar{y}$ is the mean of the true values in the evaluation set. $R^{2}$ is a common metric used for evaluating regression models in general and is frequently used to assess the quality of drug response prediction models.

#### Appendix A.1.2. Mean Squared Error (MSE)

MSE reflects the average squared deviation of the predicted values from the true values. It can be computed using the following expression:

$$MSE=\frac{1}{n}\sum_{i=1}^{n}{\left(y_{i}-\hat{y_{i}}\right)}^{2},$$

where $\hat{y_{i}}$ is a model estimate. Squaring error penalizes outliers, as a single large deviation would be amplified. It is useful for comparing models trained on the same data. However, it is challenging to assess a model quality based solely on this statistic, as it depends on the response values range.

#### Appendix A.1.3. Mean Absolute Error (MAE)

MAE describes the average deviation of the predicted values from the true values. The formula of this score is based on the $l_{1}$ metric:

$$MSE=\frac{1}{n}\sum_{i=1}^{n}\left|y_{i}-\hat{y_{i}}\right|$$

Unlike MSE, it does not specifically penalize severe outliers. As with the MSE, it is useful for comparative analysis of the models but does not provide enough information on its quality without knowledge about the values range.

#### Appendix A.1.4. Pearson Correlation Coefficient (PCC)

PCC describes the linear correlation between two sets of points. It is computed by dividing covariance between two sets of points over the product of standard deviation for each of the individual sets. For the case of 1-D points, it can be computed using the following equation:

$$\rho =\frac{n\sum \hat{y_{i}}y_{i}-\sum \hat{y_{i}}\sum y_{i}}{\sqrt{n\sum {\hat{y}}_{i}^{2}-{\left(\sum y_{i}\right)}^{2}}\sqrt{n\sum y_{i}^{2}-{\left(\sum y_{i}\right)}^{2}}}$$

#### Appendix A.1.5. Spearman Correlation Coefficient (SCC)

SCC describes the alignment of the rankings among two sets of points. It allows this score to capture non-linear components of the alignment. Correlation is defined similarly to PCC, with the only exception of using rankings of the points instead of their values:

$$r_{s}=\frac{cov(R\left(\hat{Y}\right),\;R(Y))}{\sigma (R(\hat{Y}))\sigma (R(Y))},$$

where cov(X,Y) is a covariance between X and Y, R(X) is a ranking of the set X, σ(X) is the standard deviation of X, Y is the set of the ground truth values, and $\hat{Y}$ is the set of the predicted values.

## Appendix B

It is expected that large ligand molecules may not be positioned in small, enclosed binding pockets. To validate this claim, we demonstrate that the number of missing values for docking depends on the ligand size—the number of non-hydrogen atoms that compose the ligand (Figure A1). When we use the approximate binning from [94] to group compounds into small (<20 atoms, blue), medium (20–30 atoms, yellow), and large (>30 atoms, red) ligands, it is evident that a larger molecule size corresponds to more missing values, because there are fewer viable pockets for larger ligands. Overall, small ligands correspond to 0.2% of the missing values, medium ligands constitute 4.7%, and large ligands correspond to 95.1%. This behavior is consistent with our premises.

We also explored estimating binding affinities and using them as features for drug response prediction instead of binding sores. We used the following approach to estimate binding affinities. The best docking pose for each drug molecule fitted into a receptor site was converted to a protein-ligand complex that contains an entire protein structure from the corresponding PDB structure and the drug molecule. Solvent and original ligands interacting with protein chains were removed and the drug ligand of interest was placed into the corresponding binding site. Then PRODIGY-LIG, a lightweight prediction system designed for the large-scale estimation of binding affinity for small ligands-protein complexes, was used to calculate the binding free energy for the obtained complex. PRODIGY-LIG relies on intermolecular atomic contact statistics (contact type, number, involved atoms) and electrostatics energy to calculate the binding free energy. However, the calculation of binding affinity fails for a significant portion of the drug and receptor pairs, resulting in a quite sparse binding affinity matrix with a missing rate of 83.3%. We conducted a few preliminary analyses on using the binding affinity estimates for drug response prediction instead of docking scores, and no benefit was observed in comparison with using docking scores.
